# Identification of Four Distinct Phylogenetic Groups in *Flavobacterium columnare* With Fish Host Associations

**DOI:** 10.3389/fmicb.2018.00452

**Published:** 2018-03-13

**Authors:** Benjamin R. LaFrentz, Julio C. García, Geoffrey C. Waldbieser, Jason P. Evenhuis, Thomas P. Loch, Mark R. Liles, Fong S. Wong, Siow F. Chang

**Affiliations:** ^1^Aquatic Animal Health Research Unit, United States Department of Agriculture – Agricultural Research Service, Auburn, AL, United States; ^2^Warmwater Aquaculture Research Unit, Thad Cochran National Warmwater Aquaculture Center, United States Department of Agriculture – Agricultural Research Service, Stoneville, MS, United States; ^3^National Center for Cool and Cold Water Aquaculture, United States Department of Agriculture – Agricultural Research Service, Kearneysville, WV, United States; ^4^Department of Pathobiology and Diagnostic Investigation, College of Veterinary Medicine, Michigan State University, East Lansing, MI, United States; ^5^Department of Biological Sciences, Auburn University, Auburn, AL, United States; ^6^MSD Animal Health Innovation Pte. Ltd., Singapore, Singapore

**Keywords:** *Flavobacterium columnare*, columnaris disease, 16S rRNA, genomovar, multilocus phylogenetic analysis, genetic diversity

## Abstract

Columnaris disease, caused by the Gram-negative bacterium *Flavobacterium columnare*, is one of the most prevalent fish diseases worldwide. An exceptionally high level of genetic diversity among isolates of *F. columnare* has long been recognized, whereby six established genomovars have been described to date. However, little has been done to quantify or characterize this diversity further in a systematic fashion. The objective of this research was to perform phylogenetic analyses of 16S rRNA and housekeeping gene sequences to decipher the genetic diversity of *F. columnare*. Fifty isolates and/or genomes of *F. columnare*, originating from diverse years, geographic locations, fish hosts, and representative of the six genomovars were analyzed in this study. A multilocus phylogenetic analysis (MLPA) of the 16S rRNA and six housekeeping genes supported four distinct *F. columnare* genetic groups. There were associations between genomovar and genetic group, but these relationships were imperfect indicating that genomovar assignment does not accurately reflect *F. columnare* genetic diversity. To expand the dataset, an additional 90 16S rRNA gene sequences were retrieved from GenBank and a phylogenetic analysis of this larger dataset also supported the establishment of four genetic groups. Examination of isolate historical data indicated biological relevance to the identified genetic diversity, with some genetic groups isolated preferentially from specific fish species or families. It is proposed that *F. columnare* isolates be assigned to the four genetic groups defined in this study rather than genomovar in order to facilitate a standard nomenclature across the scientific community. An increased understanding of which genetic groups are most prevalent in different regions and/or aquaculture industries may allow for the development of improved targeted control and treatment measures for columnaris disease.

## Introduction

Columnaris disease, caused by the Gram-negative bacterium *Flavobacterium columnare*, is a prevalent disease of fish due to an ability to infect most freshwater species in a range of environmental temperatures. The disease was first described in the early 1900s and clinical signs include lethargy, loss of appetite, necrotic gills, depigmented and necrotic lesions of the skin, and necrotic or frayed fins. Upon the discovery of this disease, it was suggested to be a significant pathogen due to an ability to infect numerous fish species (Davis, 1922). Currently, columnaris disease continues to impact wild (Morris et al., 2006; Scott and Bollinger, 2014; Faisal et al., 2017) and cultured species of fish worldwide such as salmonids in Finland (Suomalainen et al., 2005), channel catfish and rainbow trout in the United States (Wagner et al., 2002; Evenhuis et al., 2014), salmonids in Chile (Avendaño-Herrera et al., 2011), tilapia in Thailand (Dong et al., 2015), various species in Brazil (Barony et al., 2015), as well as other species and locations.

Although globally important, a thorough understanding of this bacterium and the disease it causes is lacking. Recent research has closed gaps in our knowledge, including host–pathogen–environment interactions (Kinnula et al., 2017), identification of functional virulence factors (Li et al., 2017), selective breeding for columnaris disease resistance (Evenhuis et al., 2015) and other important research areas (Declercq et al., 2013). It is anticipated that an increased understanding of these research areas will lead to improved methods to prevent and control columnaris disease. Currently, columnaris disease is best controlled by antibiotic or chemical use and preventative measures include management strategies and use of autogenous and licensed vaccines. While there has been some success in controlling and preventing columnaris using these measures, losses in aquaculture operations due to this disease remain substantial.

The genetic diversity among *F. columnare* isolates has been studied for several decades. Song et al. (1988) performed DNA hybridization experiments on isolates from geographically distant regions which indicated that three genetic groups existed based upon DNA homology. Toyama et al. (1996) demonstrated intraspecific nucleotide diversity in the 16S rRNA gene of isolates, and this diversity was exploited to develop a PCR-based assay for typing *F. columnare* (Triyanto and Wakabayashi, 1999). In this restriction fragment length polymorphism (RFLP) assay, a portion of the 16S rRNA gene is amplified, digested with a single restriction enzyme, and resultant DNA fragments are resolved by electrophoresis. Use of this assay on a small number of isolates categorized them into three genomovars (i.e., genetic groups), and the assay results were supported by phylogenetic analysis and DNA hybridization (Triyanto and Wakabayashi, 1999).

Following these original reports of genetic diversity, numerous studies have analyzed isolates from various locations using different molecular approaches such as random amplified polymorphic DNA (RAPD), pulsed-field gel electrophoresis (PFGE), amplified fragment length polymorphism (AFLP), single-strand conformation polymorphism (SSCP), repetitive extragenic palindromic PCR (REP-PCR), multilocus sequence analysis (MLSA), and sequencing of the 16S rRNA gene and 16S–23S intergenic spacer region. The results from these studies are strikingly similar in that most indicate at least two different groups among *F. columnare* isolates (**Table 1**). The first whole genome sequence of *F. columnare* was published in 2012 (Tekedar et al., 2012) and subsequently several other genomes have been published (Bartelme et al., 2016; Kumru et al., 2016; Zhang et al., 2016; Evenhuis et al., 2017; Kayansamruaj et al., 2017) allowing for genome based analyses. Kumru et al. (2017) performed a thorough comparative analysis of a genomovar I and II genome, representative of two genetic types of *F. columnare* (described further below). Their research suggested that the core genomes were mostly conserved, but the isolates were genetically distinct as defined by *in silico* DNA–DNA hybridization methods. Kayansamruaj et al. (2017) performed a comparative genome analysis of several genomes and their research indicated three or four groups depending on the locus used for analysis. These studies highlight the genetic diversity among *F. columnare* isolates and that phylogenetic resolution is affected by the methods used to compare isolates.

**Table 1:** Summary of publications that used molecular approaches to define the genetic diversity of *Flavobacterium columnare*.

Technique	Number of isolates analyzed	Number of groups or clusters identified	Reference
RAPD	17	3	Thomas-Jinu and Goodwin, 2004
PFGE	31	2	Soto et al., 2008
ALFP	30	4	Arias et al., 2004
	90	2	Olivares-Fuster et al., 2007a
16S-SSCP	30	2	Olivares-Fuster et al., 2007b
ISR-SSCP	90	2	Olivares-Fuster et al., 2007a
	30	2	Olivares-Fuster et al., 2007b
REP-PCR	15	4	Barony et al., 2015
MLSA	6	2	Olivares-Fuster et al., 2007a
	83	2	Ashrafi et al., 2015
	17	4	Kayansamruaj et al., 2017
16S rRNA sequence	29	4	Barony et al., 2015
	19	3	Darwish and Ismaiel, 2005
	16	3	Schneck and Caslake, 2006
	28	3	Dong et al., 2015
	6	3	Triyanto and Wakabayashi, 1999
	24	3	Kayansamruaj et al., 2017
16S–23S ISR sequence	50	2	Dong et al., 2015
	30	3	Arias et al., 2004
Genome based	9	4	Kayansamruaj et al., 2017

It is well known that development of control methods and vaccines can be complicated by genetic diversity of bacterial pathogens. In regards to vaccines, genetic diversity can result in antigenic variation that may render vaccines ineffective against heterologous isolates (Telford, 2008). The difficulty in preventing and controlling columnaris disease may reflect an inadequate understanding of *F. columnare* genetic diversity. To begin to understand the genetic diversity of *F. columnare*, we first standardized the 16S-RFLP technique (LaFrentz et al., 2014) developed by Triyanto and Wakabayashi (1999). In that study, five genomovars were described (I, II, II-B, III, and I/II) and subsequently one primer was optimized to overcome difficulties in amplifying 16S rRNA genes from all *F. columnare* isolates (LaFrentz et al., 2017). More recently, a new genomovar, II-A, was described (García et al., 2018). With this established genotyping system, the objective of the present study was to decipher *F. columnare* genetic diversity using higher resolution methods and to compare the phylogenies derived from a multilocus phylogenetic analysis (MLPA) with previously assigned genomovars.

## Materials and Methods

### Bacterial and Culture Conditions

Fifty isolates and/or genomes of *F. columnare*, originating from diverse years, geographic locations, and fish hosts, were analyzed in this study (**Tables 2, 3**). Of these, 26 (**Table 2**) were cultured from archive laboratory stocks, and extracted DNA was used to sequence genes of interest as described below. Eleven publicly available genomes and thirteen additional genomes (LaFrentz et al., unpublished; **Table 3**) were used to obtain DNA sequences from annotated genomes. The identity of isolates was confirmed by PCR using species specific primers as described by Welker et al. (2005). Frozen material from archived glycerol stocks were plated onto modified Shieh agar (LaFrentz and Klesius, 2009) plates and incubated at 28°C for 48–72 h. Single isolated colonies were then cultured in 25 mL of modified Shieh broth and incubated at 28°C for 24–36 h with shaking at 175 rpm. These cultures were used for DNA extraction.

**Table 2:** Description of *Flavobacterium columnare* isolates used in this study, including the year and fish host of isolation, geographic origin, and genomovar assignment.

Isolate	Year	Fish host	Origin	Genomovar
RCO2503	2011	Red cap oranda goldfish	Singapore	I
S15-63	2015	Channel catfish	Mississippi (United States)	I
ARS-15-4	2015	Bluegill	Alabama (United States)	I
14-051	2014	Bluehead sucker	Wyoming (United States)	I
ARS-DRB-1-10	2010	Channel catfish	Alabama (United States)	I
TI2063	2007	Tilapia	Africa	I/II
TI2429	2010	Tilapia	Thailand	II
A2502	2011	Arowana	Singapore	II
EE923	2003	Eel	China	II
BZ-5-02	2002	Nile tilapia	Brazil	II
TI472	2005	Tilapia	Malaysia	II
TI982	2003	Tilapia	Vietnam	II
TI2056	2007	Tilapia	China	II
TI1677	2005	Tilapia	Ecuador	II
TI1371	2004	Tilapia	Indonesia	II
TI1354B	2005	Tilapia	Indonesia	II
TI1690	2005	Tilapia	Honduras	II
Costa Rica 04-02-TN	2004	Tilapia	Costa Rica	II
CC1351	2004	Common carp	Indonesia	II-A
Grizzle	2000	Channel catfish	Alabama (United States)	III
AU-LMB-08-5	2008	Largemouth bass	Alabama (United States)	III
ARS-15-12	2015	Nile tilapia	Florida (United States)	III
TN-3-2012	2012	Nile tilapia	Alabama (United States)	III
ALM-05-69	2005	Freshwater drum	Alabama (United States)	III
ALM-05-140	2005	Channel catfish	Alabama (United States)	III
ALM-05-111	2005	Threadfin shad	Alabama (United States)	III

**Table 3:** Description of *Flavobacterium columnare, F. psychrophilum*, and *F. johnsoniae* genomes analyzed in this study, including the year and fish host of isolation, geographic origin, genomovar assignment, and NCBI accession number.

Isolate	Year	Fish host	Origin	Genomovar	NCBI accession
Israel	Not known	Common carp	Israel	I	Unpublished^1^
ALG-03-063	2003	Channel catfish	Alabama (United States)	I	Unpublished^1^
IA-S-4	2011	Walleye	Iowa (United States)	I	Unpublished^1^
ATCC 49512	1987	Brown trout	France	I	NC_016510^2^
CSF-298-10	2010	Rainbow trout	Idaho (United States)	I	MUAW01^3^
TC 1691	Not known	River water sample	China	I	NZ_CP018912
Pf1	Not known	Yellow catfish	China	I	NZ_CP016277^4^
F4-HK	2012	Yellow perch	Indiana (United States)	I/II	Unpublished^1^
1215	2012	Red tilapia	Thailand	I/II	MTCZ01^5^
ALG-00-530	2000	Channel catfish	Alabama (United States)	II	Unpublished^1^
AL-02-36	2002	Largemouth bass	Alabama (United States)	II	Unpublished^1^
MS-02-475	2002	Channel catfish	Mississippi (United States)	II	Unpublished^1^
C#2	Not known	Not known	Not known	II	NZ_CP015107^6^
94-081	1994	Channel catfish	Mississippi (United States)	II	NZ_CP013992^7^
CF1	2014	Striped catfish	Thailand	II	MTDC01^5^
1362	2013	Red tilapia	Thailand	II	MTDA01^5^
BZ-1-02	2002	Nile tilapia	Brazil	II	Unpublished^1^
1214	2012	Red tilapia	Thailand	II	MTCY01^5^
NK01	2014	Nile tilapia	Thailand	II	MTDD01^5^
PT-14-00-151	2000	Channel catfish	Mississippi (United States)	II-B	Unpublished^1^
FBCC-CC-12K	2013	Channel catfish	Florida (United States)	II-B	Unpublished^1^
90-106	1990	Channel catfish	Mississippi (United States)	III	Unpublished^1^
GA-02-14	2002	Rainbow trout	Georgia (United States)	III	Unpublished^1^
ARS-1	1996	Channel catfish	Alabama (United States)	III	Unpublished^1^
*F. psychrophilum* JIP02/86	1986	Rainbow trout	France	–	NC_009613^8^
*F. johnsoniae* UW101	Not known	Soil	England	–	NC_009441^9^

*^*1*^LaFrentz et al., unpublished; ^*2*^Tekedar et al., 2012; ^*3*^Evenhuis et al., 2017; ^*4*^Zhang et al., 2016; ^*5*^Kayansamruaj et al., 2017; ^*6*^Bartelme et al., 2016; ^*7*^Kumru et al., 2016; ^*8*^Duchaud et al., 2007; ^*9*^McBride et al., 2009.*

### DNA Extraction and RFLP

Bacterial genomic DNA (gDNA) was extracted from isolates using the DNeasy Blood and Tissue Kit (Qiagen) according to the manufacturer’s protocol for Gram-negative bacteria and quantified using a NanoDrop ND-1000 spectrophotometer (NanoDrop). Each isolate was assigned to genomovar as described by LaFrentz et al. (2014) using an optimized reverse primer (1500R-1; LaFrentz et al., 2017).

### Primer Design

Ashrafi et al. (2015) previously published a MLSA scheme for *F. columnare*, in which the primers were designed from the DNA sequence of a genomovar I isolate (ATCC 49512). In the present study, the same six housekeeping genes were used for a MLPA, including *trpB, gyrB, dnaK, tuf, atpA*, and *rpoD*. To ensure primer specificity for isolates assigned to different genomovars, these gene sequences were extracted from 13 draft genomes (**Table 3**; LaFrentz et al., unpublished) and analyzed. For each gene, a multiple sequence alignment was performed using CLC Genomics Workbench (version 9.5.2) and the primer binding sites designed by Ashrafi et al. (2015) were examined. Nucleotide heterogeneity was observed at the binding site of most primers (i.e., up to eight positions with differing nucleotides; data not shown); thus, new forward and reverse degenerate primers for each gene were designed using the consensus sequences at the primer binding sites used by Ashrafi et al. (2015) (**Table 4**).

**Table 4:** Sequences of primers designed for the amplification of *Flavobacterium columnare* housekeeping genes used for the MLSA and corresponding PCR cycling parameters.

Gene	Gene product	Primer name	Sequence (5′–3′)^1^	PCR cycles	Annealing temperature
16S rRNA^2^	16S ribosomal	20F	AGAGTTTGATCMTGGCTCAG	30	55°C
	RNA	1492R	GGTTACCTTGTTACGACTT		
*trpB*	Tryptophan synthase beta chain	trpB-F	TGYCATACAGGHGCDCATAA	30	45°C
		trpB-R	TKGCDCKYCCRCTTTTRAAT		
*gyrB*	DNA gyrase subunit B	gyrB-F	TACNCAYGAAGGAGGWACRC	30	51°C
		gyrB-R	GRCTMCCRTCAATATCRGCA		
*dnaK*	Chaperone protein DnaK	dnaK-F	RGCTACRGCYWCWGGRCCWA	40	51°C
		dnaK-R	AGCKRMTTTATCWGCTTCNG		
*tuf*	Translation elongation factor Tu	tuf-F	ACATGGTTACTGGTGCTGCK	35	63°C
		tuf-R	TRTGGAATGGMGTGTGACGW		
*atpA*	ATP synthase alpha chain	atpA-F	GCGTAAAGCACCAGGGGTAA	30	54°C
		atpA-R	TGGACGWACYCCWGAGTTRA		
*rpoD*	RNA polymerase sigma factor RpoD	rpoD-F	AGCWCAACGCATCAARGCWGGB	35	62°C
		rpoD-R	HGGRGCATCCATWGAYARRTGRCGW		

*^*1*^Degenerate base codes: R = A+G; Y = C+T; M = A+C; K = G+T; W = A+T; H = A+T+C; B = G+T+C; D = G+A+T; N = A+C+G+T; ^*2*^16S rRNA primer sequences from Lane (1991).*

### PCR Amplification and Sequencing

The 16S rRNA and each of the six housekeeping genes were amplified from each *F. columnare* isolate (**Table 2**) by PCR using the primers designed in this study or previously published (**Table 4**). PCR was performed with AccuPrime Pfx DNA polymerase (Invitrogen), and the final concentrations of each component in the 50 μL reaction mixture were as follows: 5 μL 10X AccuPrime Pfx reaction mix, 0.4 μM forward and reverse primer, 1 unit AccuPrime Pfx DNA polymerase and 25 ng total gDNA. PCR amplification was performed with a MyCycler thermal cycler (Bio-Rad) using a cycling protocol as follows: 1 cycle of 5 min at 95°C; 30–40 cycles of 30 s at 95°C, 20 s at 45–62°C and 45 s at 68°C; and a final cycle of 10 min at 68°C. Different numbers of PCR cycles and annealing temperatures were used for each primer pair (**Table 4**). Following amplification, PCR products were detected by subjecting 5 μL of the PCR reaction to 1.5% (w/v) agarose gel electrophoresis in Tris-acetate-EDTA (TAE buffer). Gels were precast with 1X SYBR Safe DNA gel stain (Invitrogen), and the products were visualized using ultraviolet transillumination. PCR products were purified using the QIAquick PCR Purification Kit (Qiagen) and were sequenced commercially (Eurofins Genomics) using the same primers used for PCR. Sequence reads were assembled into contigs using BioNumerics (version 6.6, Applied Maths), and sequences were verified by manually examining chromatograms. The sequences for the *gyrB, tuf, dnaK, rpoD, atpA*, and *trpB* genes were deposited into GenBank under accession numbers MG516221–MG516454 and the 16S rRNA gene sequences were deposited under accession numbers MG516944–MG516975.

### Phylogenetic Analyses

The 16S rRNA gene sequences from the 26 isolates of *F. columnare* (**Table 2**), sequences extracted from *F. columnare* genomes (**Table 3**), and sequences from *F. psychrophilum* and *F. johnsoniae* were aligned and trimmed using the Molecular Evolutionary Genetics Analysis (MEGA6) software (Tamura et al., 2013). The best nucleotide substitution model was tested in MEGA6 and the model with the lowest Bayesian Information Criterion (BIC) scores was used for the phylogenic analysis. The evolutionary relatedness of the 50 16S rRNA gene sequences was inferred using the maximum likelihood method based upon the Kimura 2-parameter model (K2+G; Kimura, 1980). Initial trees for the heuristic search were obtained by applying the neighbor-joining method to a matrix of pairwise distances estimated using the maximum composite likelihood (MCL) approach. All positions containing gaps and missing data were eliminated, leaving a total of 1,338 positions in the final dataset. The final tree was constructed from 1,000 bootstrap replicates and was rooted with *F. psychrophilum* and *F. johnsoniae*.

For MLPA, the six housekeeping gene sequences from the 26 isolates of *F. columnare* (**Table 2**), sequences extracted from annotated *F. columnare* genomes (**Table 3**), and sequences from *F. psychrophilum* and *F. johnsoniae* were aligned and trimmed using MEGA6. The six gene sequences from each individual isolate were then concatenated and used to infer the evolutionary relatedness by using the maximum likelihood method based upon the general time reversible model (GTR+G+I; Nei and Kumar, 2000). There were a total of 3,633 positions in the final dataset, the final tree was constructed from 1,000 bootstrap replicates, and was rooted with *F. psychrophilum* and *F. johnsoniae*. Additionally, a phylogenetic analysis was performed on each individual gene as indicated above.

An additional 16S rRNA gene phylogeny was performed. All available *F. columnare* 16S rRNA gene sequences were downloaded from GenBank (performed on 9/26/2017). From these, multiple sequences were removed because Basic Local Alignment Search Tool (BLASTn; Johnson et al., 2008) at the National Center for Biotechnology Information (NCBI^1^) analysis indicated they were not related to *F. columnare*. Sequences less than 1,000 nucleotides were omitted as were duplicate sequences of the same isolate. A total of 90 sequences were obtained that met these criteria and were added to the 50 sequences described above for phylogenetic analysis (Supplementary Table 1). The 140 *F. columnare* 16S rRNA gene sequences and 16S rRNA gene sequences from *F. psychrophilum* and *F. johnsoniae* were aligned and trimmed in MEGA6, whereby the evolutionary relatedness was inferred using the maximum likelihood method based upon the Kimura 2-parameter model (K2+G) as described above. There were a total of 1,061 positions in the final dataset, the final tree was constructed from 1,000 bootstrap replicates, and rooted with *F. psychrophilum* and *F. johnsoniae*.

## Results and Discussion

The objective of the present study was to decipher *F. columnare* genetic diversity using phylogenetic analyses. In order to be inclusive, a diverse panel of isolates was collected from broad geographical regions, different fish species, and across a range of years. Additionally, it was important to include isolates assigned to the six established genomovars of *F. columnare* in order to use assigned genomovars as the basis to compare phylogenies derived from DNA sequences. Therefore, 12 genomovar I, 3 genomovar I/II, 22 genomovar II, 1 genomovar II-A, 2 genomovar II-B, and 10 genomovar III isolates were included and encompassed a large degree of diversity as indicated above (**Tables 2, 3**).

**FIGURE 1:**
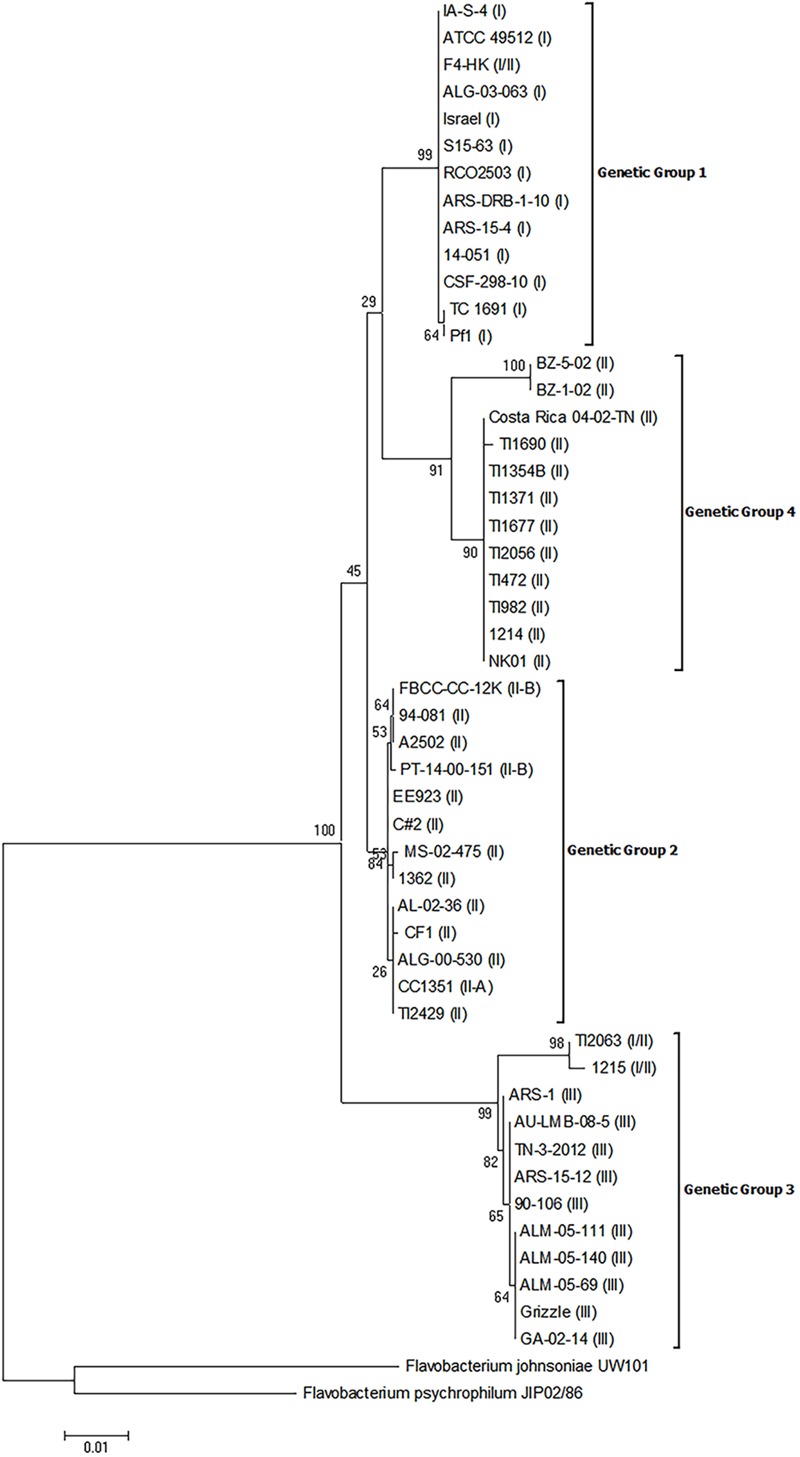
Phylogenetic relationships based on 16S rRNA gene sequences of 50 isolates of *Flavobacterium columnare*. Relatedness was inferred using the maximum likelihood method based upon the Kimura 2-parameter model (K2+G) and rooted with *F. johnsoniae* and *F. psychrophilum*. The percentage of trees in which the associated sequences clustered together in the bootstrap test (1,000 replicates) is shown next to the branches. The analysis involved 52 nucleotide sequences, all positions containing gaps and missing data were eliminated, and there were a total of 1,338 positions in the final dataset. The assigned genomovar of the isolate is in parentheses adjacent to the isolate designation.

The 16S rRNA gene sequences from these isolates were obtained by sequencing or were downloaded from publicly available and unpublished genomes. Phylogenetic analysis of these sequences resulted in the establishment of four distinct genetic groups as evidenced by bootstrap values >70 (**Figure 1**). There was an association between genomovar and genetic group, but this association was imperfect. Genetic group 1 was comprised of all genomovar I isolates and the genomovar I/II isolate, F4-HK. Genetic group 2 was comprised of a portion of the genomovar II isolates, the only genomovar II-A isolate (CC1351), and the two genomovar II-B isolates. Genetic group 3 was comprised of all genomovar III isolates and two genomovar I/II isolates. Within genetic group 3, the two genomovar I/II isolates, TI2063 and 1215, formed a separate supported group with strong bootstrap support (98). Genetic group 4 was comprised of all the remaining genomovar II isolates. Within genetic group 4, two isolates, BZ-1-02 and BZ-5-02, formed a separate group with strong bootstrap support (100). Although there were four distinct genetic groups, the presence of the two supported sub-groups within genetic groups 3 and 4, may indicate the existence of additional genetic groups. Three of the genetic groups described above contained members from more than one genomovar as assigned by 16S-RFLP. These results indicate the 16S-RFLP method may not accurately reflect the genetic diversity in the 16S rRNA gene sequences of *F. columnare*. This may be explained by the nature of this technique which only interrogates sequence variation at restriction sites. Similar results were suggested by Kayansamruaj et al. (2017). For example, an isolate assigned to genomovar I/II by RFLP could be either a genetic group 1 or 3 isolate. Additionally, the RFLP technique was unable to differentiate between isolates contained in genetic groups 2 and 4. Interestingly, all of the genomovar II isolates contained in genetic group 4 were isolates that could not be assigned to a genomovar using the standardized RFLP method as described by LaFrentz et al. (2014). The 16S rRNA genes of these isolates could not be amplified by PCR using the original typing primers (20F/1500R) due to nucleotide heterogeneity in the primer binding site in these isolates (Dong et al., 2015; LaFrentz et al., 2017). Thus, a new degenerate reverse primer was designed (1500R-1) that allowed for amplification of the 16S rRNA genes and subsequent typing to genomovar II (LaFrentz et al., 2017). This finding indicates that the nucleotide heterogeneity observed in these isolates was not likely a random occurrence in some isolates, but is an accurate reflection of their phylogeny.

**FIGURE 2:**
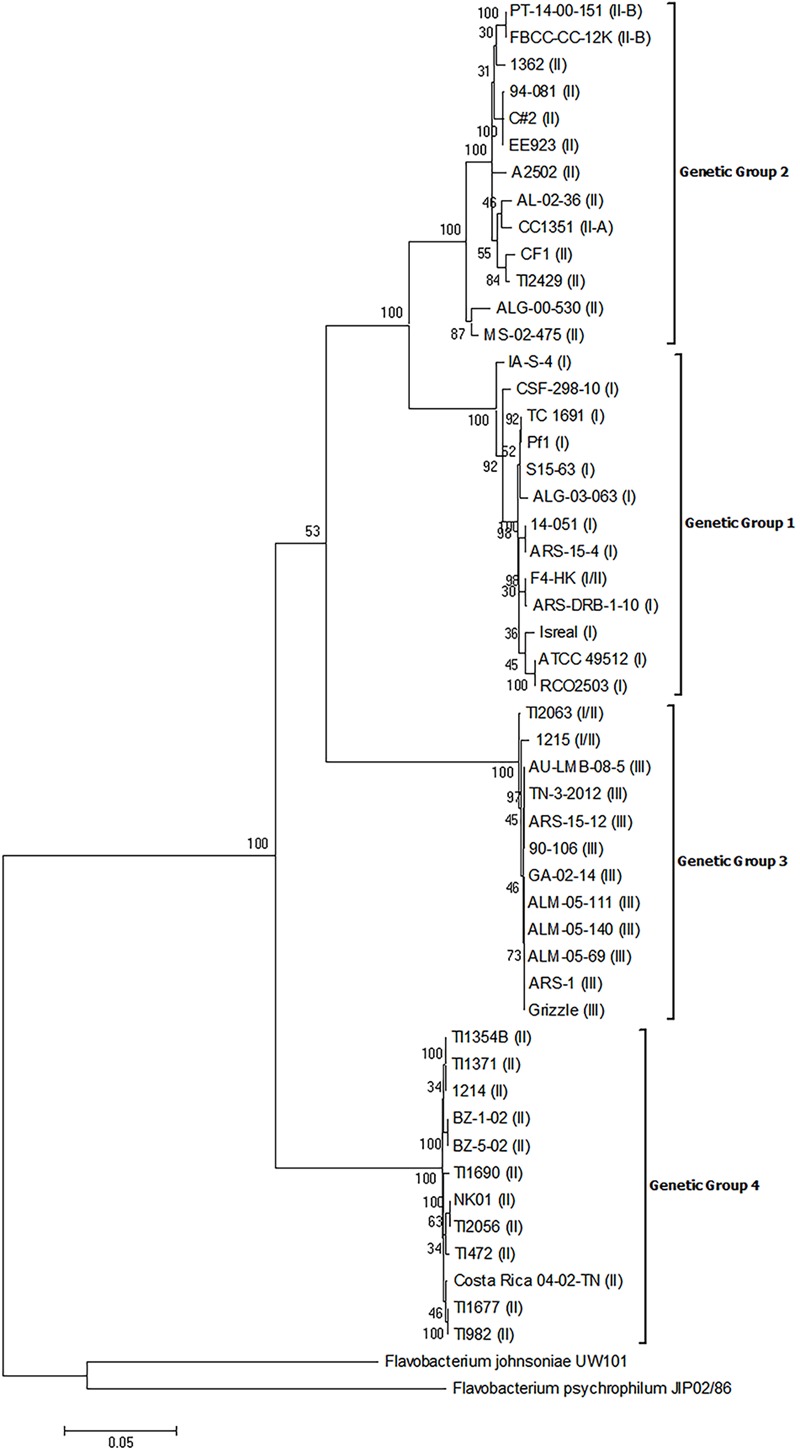
Phylogenetic relationships based on six concatenated housekeeping gene sequences of 50 isolates of *Flavobacterium columnare*. Relatedness was inferred using the maximum likelihood method based upon the general time reversible model (GTR+G+I) and rooted with *F. johnsoniae* and *F. psychrophilum*. The percentage of trees in which the associated sequences clustered together in the bootstrap test (1,000 replicates) is shown next to the branches. The analysis involved 52 nucleotide sequences, all positions containing gaps and missing data were eliminated, and there were a total of 3,633 positions in the final dataset. The assigned genomovar of the isolate is in parentheses adjacent to the isolate designation.

The MLPA analysis of the concatenated housekeeping gene sequences also resulted in the establishment of four distinct genetic groups (**Figure 2**) and there was 100% agreement in the placement of isolates into each genetic group between the analysis of the 16S rRNA genes and the MLPA. Moreover, each genetic group was robustly supported as distinct from all other genetic groups as evidenced by bootstrap values >70. Additionally, there was a greater level of sequence heterogeneity in the MLPA analysis compared to the 16S rRNA phylogeny, as expected. However, the supported subgroups within genetic groups 3 and 4 that were present in the 16S rRNA gene phylogeny were not present in the MLPA phylogeny, which suggests these subgroups are not unique genetic groups.

Phylogenetic analyses were also performed using each of the six individual gene sequences used in the MLPA. Analysis of the *gyrB, tuf*, and *dnaK* gene sequences also resulted in the formation of four distinct genetic groups with bootstrap values >70 (Supplementary Figures 1–3). Analysis of the *dnaK* gene resulted in the most robustly supported tree delineating the four genetic groups (bootstrap values of 99–100), and all basal nodes within the dendrogram were also highly supported (bootstrap values of 99–100). Thus the *dnaK* gene may represent an optimal locus to assign *F. columnare* isolates to a genetic group. Analysis of the *rpoD* gene sequences resulted in robust support for each genetic group; however, three genetic group 1 isolates (CSF-298-10, ALG-03-063, and IA-S-4) could not be ascribed to this genetic group via this locus (Supplementary Figure 4). Phylogenetic analysis of the *atpA* gene sequences resulted in robust support for each genetic group; however, two genetic group 2 isolates (MS-02-475 and ALG-00-530) formed a separate clade (Supplementary Figure 5). Phylogenetic analysis of the *trpB* gene sequences resulted in robust support for genetic groups 3 and 4; however, the topology was mostly unresolved for genetic groups 1 and 2 (Supplementary Figure 6). This is likely due to the high allelic diversity previously observed in this gene following an analysis of genomovar I (i.e., genetic group 1) isolates (Ashrafi et al., 2015).

**FIGURE 3:**
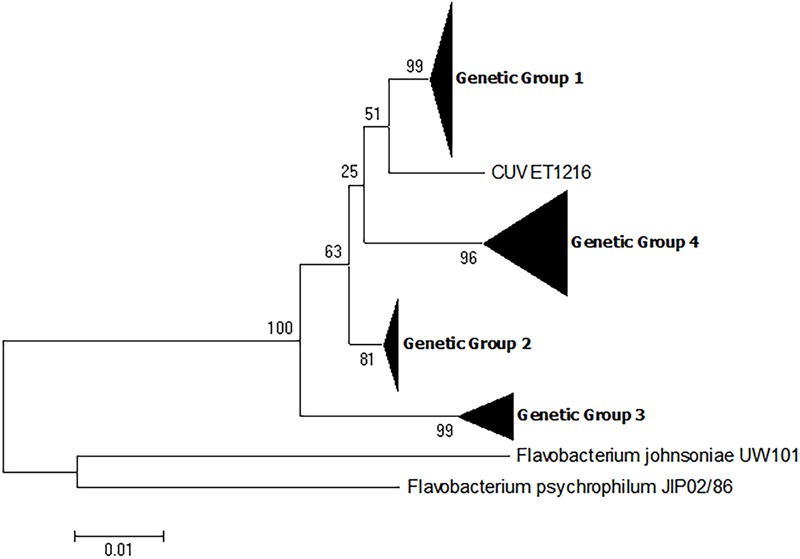
Phylogenetic relationships based on 16S rRNA gene sequences of 140 isolates of *Flavobacterium columnare*. Relatedness was inferred using the maximum likelihood method based upon the Kimura 2-parameter model (K2+G) and rooted with *F. johnsoniae* and *F. psychrophilum*. The percentage of trees in which the associated sequences clustered together in the bootstrap test (1,000 replicates) is shown next to the branches. The analysis involved 142 nucleotide sequences, all positions containing gaps and missing data were eliminated, and there were a total of 1,061 positions in the final dataset. See Supplementary Table 1 for individual isolates falling into each genetic group.

The phylogenetic analyses provided strong support for the presence of four genetic groups among the *F. columnare* isolates described in this study. To expand the dataset, all 16S rRNA gene sequences deposited into GenBank meeting the criteria defined in the materials and methods (*n* = 90) were included with the 50 sequences of the present study for phylogenetic analysis. Similar to the previous phylogenetic analyses, analysis of these sequences resulted in the establishment of four distinct genetic groups as evidenced by bootstrap values >70. (**Figure 3** and Supplementary Table 1). All 16S rRNA sequences retrieved from GenBank fell into the four genetic groups with the exception of one isolate, CUVET1216. However, it is unknown whether the sequence of this isolate is divergent from the four genetic groups or if there may have been sequencing errors. This isolate was recovered from red tilapia in Thailand (Dong et al., 2015), and the majority of isolates from this source were assigned to genetic group 4. It is possible that there is additional genetic diversity in *F. columnare*; however, these results suggest that the isolates selected for analysis in the present study are representative of the currently known genetic diversity in *F. columnare*.

Although the phylogenetic analyses identified four genetic groups, there were differences in the topologies depending upon the marker used (i.e., 16S rRNA, MLPA, or individual housekeeping genes). For example the phylogenetic analysis of the 16S rRNA gene sequences placed genetic group 3 isolates as a sister group to all other genetic groups, while the analysis of the concatenated housekeeping genes placed genetic group 4 as the sister group (**Figures 1, 2**). Kayansamruaj et al. (2017) also observed a similar difference in branching order when the 16S rRNA and the same concatenated housekeeping genes were used for phylogenetic analyses of *F. columnare*. These authors also generated a whole genome phylogenetic tree based on concatenated single nucleotide polymorphisms (SNPs) of the core genomes and the resultant tree topology was different from both the 16S rRNA and multilocus sequence phylogenies. Additionally, there were variable topologies upon phylogenetic analyses of the individual housekeeping genes in the present study (Supplementary Figures 1–6). Ashrafi et al. (2015) also observed variable topologies for *F. columnare* between phylogenetic analyses of the individual six housekeeping genes. One potential reason for these differences may be the existence of multiple copies of the 16S rRNA gene in *F. columnare*, and/or polymorphisms among the multiple rrn copies. LaFrentz et al. (2014) demonstrated that in the 1,254 bp region of the 16S rRNA gene used for the 16S-RFLP of *F. columnare*, some isolates exhibited up to 10 SNPs between the multiple copies of this gene. Since most 16S rRNA sequences are obtained from direct sequencing of PCR products, the resultant sequence is a consensus from the multiple copies and use of these may impact phylogenetic analyses. Another potential reason for these differences may be due to different evolutionary histories of the housekeeping genes compared the 16S rRNA gene. For instance comparison of the 16S rRNA genes between representative isolates from each genetic group show a percent identity >96%, while for the sequences of the housekeeping genes, the percent identities were as low as 85% (data not shown). The use of low variation and higher variation genes likely contribute to the topology inconsistencies observed between markers. Due to the observation of variable topologies following phylogenetic analyses of different markers in the present and previous studies, future research should critically analyze whole genome sequences and construct genome based phylogenies to gain a better understanding of how these genetic groups were established as was recently published with *F. psychrophilum* (Duchaud et al., 2018).

Study findings highlight the existence of four phylogenetically distinct genetic groups within the species *F. columnare*. Since the results demonstrate that 16S-RFLP and genomovar assignment does not accurately reflect the genetic diversity in *F. columnare*, we propose that instead of genomovars, isolates be assigned to the genetic groups defined in this study. This can be achieved by sequencing the 16S rRNA gene or the *dnaK* gene (**Table 4**) and subsequent phylogenetic analysis that includes both the isolate(s) of interest and sequences belonging to each of the four genetic groups. This can be laborious and time consuming, so our laboratory is currently developing a specific multiplex PCR that can assign an unknown *F. columnare* isolate to genetic group in a single PCR. This assay will facilitate standard nomenclature for the genetically disparate groups within *F. columnare* across the scientific community, a matter of importance given the worldwide distribution of this important fish pathogen and the need for universally comparable genetic typing schemes. Recent analyses of *F. columnare* genomes further illustrate the importance of this. For example, Kumru et al. (2016, 2017) determined the average nucleotide identity (ANI) between a genetic group 1 isolate (ATCC 49512) and a genetic group 2 isolate (94-081) to be 90.71% and suggested that the two isolates may be classified as different species based upon the <95% ANI cut-off for species delineation (Goris et al., 2007). Kayansamruaj et al. (2017) recently published and analyzed five *F. columnare* genomes and suggested that species designation of *F. columnare* may need to be amended. Their phylogenetic analyses are consistent with those of the present study and identified four distinct clusters. Additionally, they determined the digital DNA–DNA hybridization (dDDH) values between the genomes of isolates they sequenced as well as publicly available *F. columnare* genomes. In the context of this study, comparison of the genomes between isolates corresponding to the different genetic groups 1, 2, 3, and 4, resulted in dDDH values of less than 43% (Kayansamruaj et al., 2017). These dDDH values were well below the <70% cut-off for species delineation (Auch et al., 2010). Therefore, based solely upon analysis of DNA sequences, the genetic groups identified in this study may represent four different species of bacteria; *F. columnare* (genetic group 1) and three additional species (genetic groups 2, 3, and 4). Additional research is needed to determine whether these are unique species of bacteria or different genetic types of the same species of bacteria.

**FIGURE 4:**
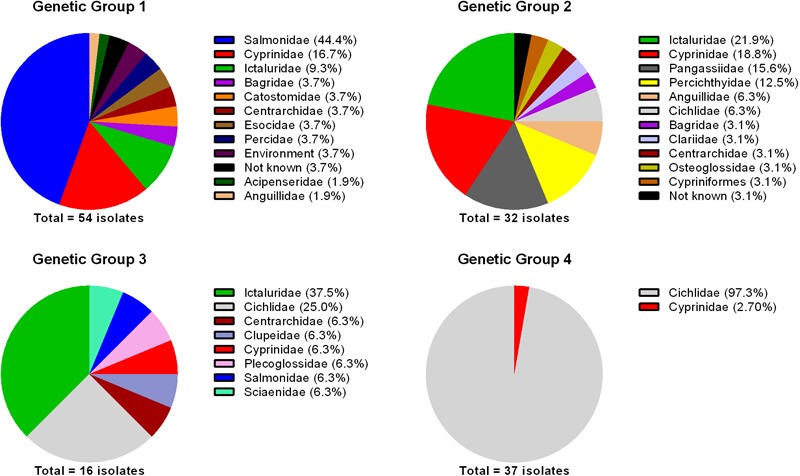
Percentage of *Flavobacterium columnare* isolates in each genetic group recovered from fish species in the families indicated. The number of isolates included in the analysis is indicated below the pie charts.

It is imperative to determine whether there is any biological relevance to the genetic diversity revealed from MLPA analyses. To address this, historical data (species and family of fish, and country of origin) for each of the 140 isolates included in the 16S rRNA phylogeny were retrieved from GenBank and/or publications (Supplementary Table 1). Although this dataset may be limited and biased based upon publication of *F. columnare* 16S rRNA gene sequences, some interesting associations were found between genetic groups, the family of fish from which the isolate was recovered, and country of origin. For genetic group 1, the majority of isolates were recovered from the families Salmonidae (44.4%), Cyprinidae (16.7%), and Ictaluridae (9.3%; **Figure 4**) in North and South America, Asia, and Europe. The association between genomovar I isolates (i.e., genetic group 1) and salmonids was previously noted (LaFrentz et al., 2012), and in this study all but one isolate recovered from salmonids were genetic group 1 (Supplementary Figure 7). Evenhuis and LaFrentz (2016) determined the virulence of genetic group 1, 2, and 3 isolates in rainbow trout (*Oncorhynchus mykiss*) and the results clearly demonstrated genetic group 1 isolates were more virulent than isolates in genetic groups 2 and 3. Salmonids are cold water fish species, and early research demonstrated that genomovar I isolates (i.e., genetic group 1) were the only isolates capable of growth at 15°C (Triyanto and Wakabayashi, 1999), which may explain this association. Evenhuis and LaFrentz (2016) also showed that challenge of rainbow trout with some genomovar III (i.e., genetic group 3) isolates resulted in moderate mortality (up to 30%). The one isolate recovered from a salmonid that did not belong to genetic group 1 belonged to genetic group 3 (Supplementary Figure 7). The virulence studies of Evenhuis and LaFrentz (2016) may provide some basis for the isolation and virulence of a genetic group 3 isolate from infected rainbow trout.

For genetic group 2, the majority of isolates were recovered from the families Ictaluridae (21.9%), Cyprinidae (18.8%), Pangasiidae (15.6%), and Percichthyidae (12.5%; **Figure 4**) from North America and Asia. For genetic group 3, which contained the lowest number of isolates (*n* = 16), the majority of isolates were recovered from the families Ictaluridae (37.5%) and Cichlidae (25%; **Figure 4**) from North America, Asia, and Africa. Shoemaker et al. (2008) tested the virulence of several genomovar I (i.e., genetic group 1) and genomovar II (i.e., genetic group 2) isolates in channel catfish (*Ictalurus punctatus*). The research demonstrated that genetic group 2 isolates were extremely virulent, with mortality greater than 60% for all isolates tested and the genetic group 1 isolates were less virulent. However, LaFrentz et al. (2014) discovered that some of the genetic group 1 isolates tested were misclassified and were actually genetic group 3. Therefore, in that study, only two genetic group 1 isolates (ATCC 23463 and MS-02-463) were tested and these did not cause any mortality. The genetic group 3 isolates tested were virulent and resulted in up to 46% mortality (Shoemaker et al., 2008). This virulence data tends to agree with the finding that the majority of isolates recovered from fish in the family Ictaluridae were genetic groups 2 and 3 (**Figure 4** and Supplementary Figure 7). Additional studies are needed to determine the virulence of genetic group 1 isolates in fish of the family Ictaluridae because they have been recovered from epizootics caused by these isolates (Supplementary Figure 7).

For genetic group 4, all isolates, with the exception of one were recovered from tilapia (*Oreochromis* sp.) in the family Cichlidae (97.3%; **Figure 4**). These isolates were recovered from infected fish in South America, Central America, and Asia. Shoemaker and LaFrentz (2015) determined the virulence of isolates from genetic groups 1, 2, and 3 in hybrid tilapia and demonstrated that there was no association between virulence and genetic group; some isolates from each genetic group were capable of causing moderate to high mortality. Unfortunately, the virulence of genetic group 4 isolates were not tested in this research. Since genetic groups 2, 3, and 4 have been recovered from infected tilapia (**Figure 4** and Supplementary Figure 7) and Shoemaker and LaFrentz (2015) demonstrated genetic group 1 isolates are virulent in this species, it would be of interest to determine if genetic group 4 isolates exhibit higher virulence in tilapia. Enhanced knowledge on this may provide support for the apparent association between genetic group 4 isolates and tilapia. Also of interest in regards to genetic group 4 isolates was the finding that they have only been recovered from Asia and South/Central America. If these isolates do have an association with tilapia, it may be possible that these isolates were spread to South and Central America from Asia with the movement of tilapia for aquaculture production. Our laboratory has analyzed over 200 *F. columnare* isolates, mostly of United States origin, and thus far none have been assigned to genetic group 4.

The results from this research establish the existence of four phylogenetically distinct genetic groups within the species *F*. *columnare* and demonstrate that 16S-RFLP and genomovar assignment does not accurately reflect this genetic diversity. Therefore, we propose that isolates be assigned to the genetic groups defined in this study rather than genomovar to facilitate a standard nomenclature across the scientific community. Analysis of the historical data of the isolates utilized indicates there is biological relevance to the genetic diversity identified. Thus, increased knowledge on the genetic groups of *F. columnare* that are most prevalent in different regions and/or aquaculture industries may allow for the development of better targeted control and treatment measures for columnaris disease.

## Author Contributions

BL conceived the research, analyzed the DNA sequence data, performed the phylogenetic analyses, interpreted the data, and prepared the manuscript. JG managed the bacterial isolates, performed DNA extraction, PCR, and RFLP. GW and JE assisted with sequencing and data analysis. TL and ML assisted with the interpretation of the phylogenetic data. FW and SC assisted with bacterial isolate management. All authors contributed to manuscript revision, and read and approved the submitted version.

## Conflict of Interest Statement

The authors declare that the research was conducted in the absence of any commercial or financial relationships that could be construed as a potential conflict of interest.

## Footnotes

1 http://blast.ncbi.nlm.nih.gov
