# Supplementary material for: Identification of Four Distinct Phylogenetic Groups in Flavobacterium columnare With Fish Host Associations
Source: Front Microbiol. 2018 Mar 13;9:452. doi: 10.3389/fmicb.2018.00452 (PMC5859164; doi:10.3389/fmicb.2018.00452)
Supplement: Supplementary file 2 [file Data_Sheet_1.docx]

***Supplementary Material***

**Identification of four distinct phylogenetic groups in *Flavobacterium columnare* with fish host associations**

**Benjamin R. LaFrentz^1*^, Julio C. García^1^, Geoffrey C. Waldbieser^2^, Jason P. Evenhuis^3^, Thomas P. Loch^4^, Mark R. Liles^5^, Fong S. Wong^6^, Siow F. Chang^6^**

***Correspondence:** *Dr. Benjamin R. LaFrentz; benjamin.lafrentz@ars.usda.gov*

**

**

Supplementary Figure 1│Phylogenetic relationships based on *gyrB* gene sequences of 50 isolates of *Flavobacterium columnare*. Relatedness was inferred using the maximum likelihood method based upon the Tamura 3-parameter model (T92+I) and rooted with *F. johnsoniae* and *F. psychrophilum*. The percentage of trees in which the associated sequences clustered together in the bootstrap test (1000 replicates) is shown next to the branches. The analysis involved 52 nucleotide sequences, all positions containing gaps and missing data were eliminated, and there were a total of 635 positions in the final dataset.





Supplementary Figure 2│Phylogenetic relationships based on *tuf* gene sequences of 50 isolates of *Flavobacterium columnare*. Relatedness was inferred using the maximum likelihood method based upon the general time reversible model (GTR+I) and rooted with *F. johnsoniae* and *F. psychrophilum*. The percentage of trees in which the associated sequences clustered together in the bootstrap test (1000 replicates) is shown next to the branches. The analysis involved 52 nucleotide sequences, all positions containing gaps and missing data were eliminated, and there were a total of 625 positions in the final dataset.





Supplementary Figure 3│Phylogenetic relationships based on *dnaK* gene sequences of 50 isolates of *Flavobacterium columnare*. Relatedness was inferred using the maximum likelihood method based upon the Tamura 3-parameter model (T92+G) and rooted with *F. johnsoniae* and *F. psychrophilum*. The percentage of trees in which the associated sequences clustered together in the bootstrap test (1000 replicates) is shown next to the branches. The analysis involved 52 nucleotide sequences, all positions containing gaps and missing data were eliminated, and there were a total of 661 positions in the final dataset.





Supplementary Figure 4│Phylogenetic relationships based on *rpoD* gene sequences of 50 isolates of *Flavobacterium columnare*. Relatedness was inferred using the maximum likelihood method based upon the Tamura 3-parameter model (T92+G+I) and rooted with *F. johnsoniae* and *F. psychrophilum*. The percentage of trees in which the associated sequences clustered together in the bootstrap test (1000 replicates) is shown next to the branches. The analysis involved 52 nucleotide sequences, all positions containing gaps and missing data were eliminated, and there were a total of 391 positions in the final dataset.





Supplementary Figure 5│Phylogenetic relationships based on *atpA* gene sequences of 50 isolates of *Flavobacterium columnare*. Relatedness was inferred using the maximum likelihood method based upon the general time reversible model (GTR+G) and rooted with *F. johnsoniae* and *F. psychrophilum*. The percentage of trees in which the associated sequences clustered together in the bootstrap test (1000 replicates) is shown next to the branches. The analysis involved 52 nucleotide sequences, all positions containing gaps and missing data were eliminated, and there were a total of 676 positions in the final dataset.





Supplementary Figure 6│Phylogenetic relationships based on *trpB* gene sequences of 50 isolates of *Flavobacterium columnare*. Relatedness was inferred using the maximum likelihood method based upon the Tamura 3-parameter model (T92+G+I) and rooted with *F. johnsoniae* and *F. psychrophilum*. The percentage of trees in which the associated sequences clustered together in the bootstrap test (1000 replicates) is shown next to the branches. The analysis involved 52 nucleotide sequences, all positions containing gaps and missing data were eliminated, and there were a total of 645 positions in the final dataset.





Supplementary Figure 7│ Percentage of each genetic group of *Flavobacterium columnare* isolates recovered from different fish families. The number of isolates included in the analysis is indicated below the pie charts.
